# Multiple symptomatic giant coronary aneurysms

**DOI:** 10.1002/ccr3.5701

**Published:** 2022-04-05

**Authors:** Damir Vukomanovic, Samuel Unzek, William Reichert, Farouk Mookadam

**Affiliations:** ^1^ The University of Arizona College of Medicine Phoenix Arizona USA

**Keywords:** angiography, CABG, coronary aneurysm, kawasaki

## Abstract

We describe a rare case of coronary artery aneurysms due to Kawasaki disease in an elderly patient. Our case presents multiple giant coronary artery aneurysms affecting the left coronary system which is less common compared with right coronary aneurysms. Giant coronary artery aneurysms are uncommon; even more rare is their association with ischemic symptoms, and treatment can be challenging. We describe a case of symptomatic multiple coronary artery aneurysms with symptom relief after coronary artery bypass grafting in an elderly patient.

## Graphical abstract

A serious sequalae of Kawasaki disease is the development of coronary artery aneurysms. Giant aneurysms are rare, and management can be difficult. Asymptomatic coronary aneurysms are managed expectantly with antiplatelet agents. For symptomatic aneurysms, percutaneous intervention favors smaller‐sized aneurysms, whilst surgery is a more effective option for giant coronary aneurysms.

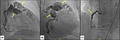

## 1 INTRODUCTION

Kawasaki disease was first recognized and described in 1961 by Tomisaku Kawasaki with approximately 85% of cases occurring in the pediatric population.^1^ It is an acute systemic vasculitis of medium‐sized vessels with a diagnostic pentad of high‐grade fever, mucositis, cervical lymphadenopathy, extremity edema, and rash.^2^ The most serious complication of the disease is progression of the coronary artery vasculitis to aneurysm formation.

## 2 CASE REPORT

A 69‐year‐old female patient presented with exertional chest discomfort relieved by nitroglycerine, typical of coronary insufficiency. Her past medical history includes Kawasaki disease (KD) that was diagnosed at age 50 after investigations for an episode of retinal artery occlusion. She had since been managed conservatively with anticoagulation with warfarin and risk factor modification.

**FIGURE 1:**
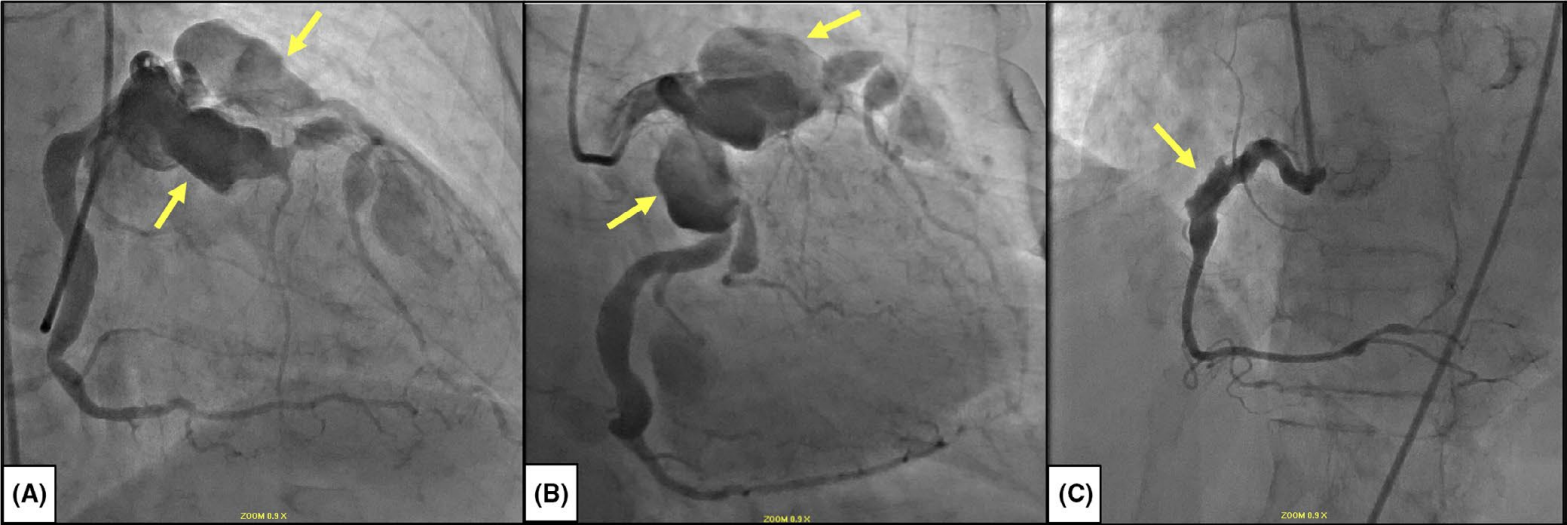
Coronary angiography. (A) RAO cranial and (B) RAO caudal views with two giant 30mm coronary artery aneurysms of the LAD and LCx coronary arteries (arrows). (C) LAO cranial view depicting a 12mm coronary artery aneurysm at the proximal RCA (arrow). *RAO*: *right anterior oblique*; *LAO*: *left anterior oblique*; *LAD*: *left anterior descendingLCx*: *left circumflex*; *RCA*: *right coronary artery*

The current clinical presentation prompted risk stratification with stress testing that demonstrated anterior wall ischemia. Subsequent coronary angiogram revealed a left anterior descending coronary artery dilation measuring 30 mm in the segment with no significant luminal stenosis (Figure 1A), a heavily calcified aneurysm of 30 mm at the ostium of the left circumflex artery (Figure 1B), and a 12 mm dilation in the proximal portion of the right coronary artery that was free of angiographically significant stenosis (Figure 1C). Additional smaller aneurysms were visualized in the proximal first obtuse marginal and mid‐second obtuse marginal arteries.

This patient was found to have new onset of unstable angina in the setting of multiple giant coronary aneurysms, with no evidence of significant atherosclerotic plaque. She had been on thromboembolic prophylaxis with warfarin maintained at a therapeutic international normalized ratio of 2–3 since the time of KD diagnosis. She was also placed on antiplatelet agents but continued to be symptomatic with angina. In the setting of optimal medical management and ongoing symptoms with known coronary aneurysms, coronary artery bypass surgery was recommended. As a result, a 3‐vessel coronary artery bypass grafting (CABG) was successfully performed with a left internal mammary grafting to the mid‐left anterior descending artery, saphenous vein graft to the posterior‐lateral ventricular branch of the distal left circumflex artery, and saphenous vein graft to the distal right coronary artery. At 6 months and 1 year postoperative follow‐up, the patient reports marked improvement with complete resolution of her symptoms after CABG, now NYHA class 1.

## 3 DISCUSSION

Coronary artery aneurysm as a sequel in Kawasaki Disease is seen in approximately 25% of cases, usually occurring at the proximal segments of the coronary artery, ostia or at the bifurcation points.^3^, ^4^ Coronary aneurysms are often classified by their maximal internal diameter. A diameter greater than 1.5 times or 50% that of the largest segment of the coronary artery meets diagnostic criteria for aneurysm.^5^ Giant coronary aneurysms do not have a universal definition but are typically >8 mm in the pediatric population or >20 mm in adults, and/or >4 times the normal reference coronary artery diameter.^6^ In adults, coronary aneurysms are often caused by atherosclerosis, in contrast to Kawasaki disease in children, and the incidence lies between 1.5% and 5% with a male predominance.^7^ The right coronary artery is affected approximately 40% of the time, and complications often include thrombosis, embolization, rupture, and vasospasm.^8^, ^9^, ^10^

Medical management of coronary aneurysms includes antiplatelet monotherapy with aspirin for small coronary aneurysms, and dual antiplatelet therapy with aspirin and a P2Y12‐inhibitor for moderate‐sized coronary aneurysms.^11^ Because giant and rapidly expanding coronary aneurysms carry an even greater risk for thrombosis, antiplatelet therapy in conjunction with an anticoagulant is often recommended.^11^ Ultimately, these aneurysms may require revascularization, either percutaneously or surgically. There is limited outcome data regarding percutaneous coronary intervention (PCI) for giant coronary aneurysms, but it does carry an increased risk for wall rupture, distal embolization, and risk of stent thrombosis.^12^, ^13^ For smaller coronary aneurysms between 6 and 10 mm, PCI with polytetrafluoroethylene (PTFE)‐covered stents appears to be an appropriate and safe option but has been associated with a high restenosis rate when used for aneurysms greater than 10 mm.^14^ Surgical intervention with CABG is often utilized for severely symptomatic or high rupture risk aneurysms, most carrying obstructive disease as well.^15^

## 4 CONCLUSION

Giant coronary aneurysms are rare. As a sequalae of Kawasaki disease, patients may develop coronary aneurysms and present with ischemic symptoms. Management may be challenging, and PCI versus CABG should be discussed. PCI may be feasible for smaller‐sized aneurysms; however, CABG appears to be an effective modality for giant coronary aneurysms. This unique case highlights the striking image, clinical reasoning, and complex decision‐making in the management of giant coronary aneurysms in an adult with Kawasaki disease.

## CONFLICTS OF INTEREST

The authors have no conflicts of interests or financial disclosures.

## AUTHOR CONTRIBUTIONS

D.V. conceptualized and prepared the manuscript, reviewed the literature, made revisions, and submitted the report. S.U. and W.R. edited drafts for clarity, accuracy, and clinical content, and provided the images. F.M. reviewed cited literature and revised drafts of all sections for important intellectual content. All authors were involved with creation of the final manuscript and approved the final report.

## CONSENT

Written informed consent was obtained from the patient to publish this report in accordance with the journal's patient consent policy.

## Data Availability

Data sharing is not applicable to this article as no new data were created or analyzed in this study.
